# Abobotulinum Toxin A in the Treatment of Chronic Low Back Pain

**DOI:** 10.3390/toxins8120374

**Published:** 2016-12-15

**Authors:** Duarte Machado, Aditya Kumar, Bahman Jabbari

**Affiliations:** Department of Neurology, Yale University School of Medicine, New Haven, CT 06510, USA; duarte.machado@yale.edu (D.M.), aditya.kumar@yale.edu (A.K.)

**Keywords:** low back pain, abobotulinum toxin A, botulinum neurotoxin, randomized controlled trial

## Abstract

Chronic low back pain is a debilitating condition with a complex and multifactorial pathophysiology. Botulinum neurotoxins (BoNTs) have strong analgesic effects, as shown in both animal models of pain and in human beings. A randomized, double-blind, placebo-controlled, parallel format study to investigate the efficacy of abobotulinum toxin A (aboA) in chronic low back pain was conducted. The study cohort consisted of 18 patients who received 100 units of aboA into each of the five lumbar extensor spinae muscles unilaterally or bilaterally (total dose 500 to 1000 units), and 19 who received normal saline of the same volume. The level of pain and quality of life were assessed using the visual analogue scale (VAS) and three questionnaires including the Oswestry Low Back Pain Disability Questionnaire (OLBPDQ). Patients’ perception of improvement was recorded via patient global impression of change (PGIC). The primary outcome measure, the proportion of responders with VAS of <4 at 6 weeks, was not met, but the data was significantly in favor of aboA at 4 weeks (*p* = 0.008). The total Oswestry score representing quality of life improved in the aboA group compared to the placebo group (*p* = 0.0448). Moreover, significantly more patients reported their low back pain as “much improved” in the abobotulinum toxin A group (0.0293).

## 1. Introduction

Low back pain is the leading cause of disability in the United States and worldwide [1,2]. Chronic low back pain is defined as pain lasting for more than 12 weeks [3]. Between 2–7% of patients with acute low back pain develop chronic low back pain [4]. The direct and indirect cost of low back pain to the US economy has been estimated to be between 12 and 90 billion dollars per annum, but the real cost may be considerably higher than these figures [5].

A multitude of pathologies including, but not limited to, chronic disc disease, lumbar spinal stenosis and osteoarthritis can be responsible for chronic low back pain, and so management is extremely challenging. Relief is often partial and side effects of analgesic and opioid medications hinder their chronic use. New and more efficacious modes of treatment for chronic low back pain are being investigated, such as the use of ultrasound and high intensity laser therapy [6]. Botulinum neurotoxins (BoNTs) may be a potential therapeutic option, having shown efficacy in animal pain models [7,8]. They are 150 kDa bacterial AB protein toxins produced by *Clostridium botulinum* [9]. BoNTs consist of three primary domains, two of which enable binding to axon terminals and their internalization, while the third has a metalloprotease that inhibits the release of neurotransmitters [10]. In addition to the well-known anti-spasmodic effect of these agents via blockade of acetylcholine release, an analgesic effect seems to be due to peripheral inhibition of pain transmitters and modulators (glutamate, calcitonin gene related peptide, substance P, etc.) [11,12,13,14]. More recent data suggest a direct central analgesic effect via migration of the toxin molecule to the central nervous system [15,16], although proof of the toxin’s central effect is still pending in humans. Chronic migraine is now an FDA-approved indication for the use of onabotulinum toxin A (onaA, *Botox*). Randomized, class I [17] placebo-controlled studies have recently shown efficacy of BoNTs in post-traumatic, post-herpetic, and trigeminal neuralgias [18,19,20].

In this communication, we report the result of an investigation on the effects of abobotulinum toxin A (aboA, *Dysport*) in chronic low back pain.

## 2. Results

The study stopped due to relocation of the PI after enrolling 43 patients. Of these, 6 were lost in follow up (3 in placebo and 3 in aboA group). The final analysis was performed on 37 subjects (18 aboA and 19 placebo). The mean age of participants was 49.2 years in the placebo (range 27–69) and 52.5 years (range 18–78) in the neurotoxin group. The baseline characteristics of these patients are depicted in Table 1. Magnetic resonance studies of the lumbar spine did not reveal any acute pathology or surgically correctable lesions in any of the study subjects.

The primary outcome of this study was the proportion of responders with a visual analogue scale (VAS) of <4 at 6 weeks. At 6 weeks, 5 subjects in the toxin group and 3 subjects in the placebo group (28% and 16%) met this criterion (*p* = 0.4470). However, more patients in the aboA group (9 out of 18) showed improvement of at least 2 grades on the VAS score compared to the placebo group (4 out of 19) at 6 weeks (*p* = 0.0911). Furthermore, at 4 weeks, 7 of 18 (39%) subjects in the aboA group and 4 of 19 subjects (21%) in the placebo group demonstrated a VAS score of <4 (*p* = 0.0084). With regard to the secondary outcomes, the total score in the Oswestry questionnaire (representing functionality and quality of life) improved in 10 of 18 subjects (55.5%) in the aboA group, but only in 4 of 19 (22.2%) subjects in the placebo group (*p* = 0.0448). Eight of 18 (44.4%) subjects in the aboA group and 2 of 19 subjects in the placebo group (10.5%) expressed their pain as “much improved” in the patient global impression of change scale (PGIC), (*p* = 0.0293). Improvement (≥2 grades) of quality of life in the ACPA scale was reported by 5 patients in the aboA group and 1 patient in the placebo group (*p* = 0.0897). No significant differences between the two groups were noted in relation to the total score in the short form-36 (SF-36) scale. A summary of the results is presented in Table 2.

There were no serious side effects during the duration of the study. Three patients in the toxin group and 2 patients in placebo group developed local pain at the site of injection which lasted for a few days (*p* = 0.6390).

## 3. Discussion

Low back pain has a complex and multifactorial etio-pathogenesis. Primary diseases of the intervertebral disc and the vertebrae can initiate a cascade of events by compression of neural tissue and development of local inflammation that leads to the accumulation of pain-inducing neurotransmitters, paraspinal muscle spasm, overgrowth of sympathetic fibers around dorsal root ganglia, and central and peripheral sensitization of nociceptive neural tissue. The pain may even be ischemic in nature due to a paraspinal compartment syndrome [21,22,23,24].

Botulinum neurotoxins can have an analgesic effect by acting on several pain inducing mechanisms which have been tested in animal studies. They are known to inhibit the release of a number of neurotransmitters, including acetylcholine, substance P, calcitonin gene-related peptide, bradykinin, and glutamate, from presynaptic vesicles via action on soluble *N*-ethylmaleimide-sensitive factor attachment protein receptor (SNARE). Injection of BoNTs in rats has been shown to significantly reduce both neurogenic inflammation and pain induced by formalin, capsaicin, and allyl isothiocyanate (the active component of mustard oil and wasabi) along with a reduction in the local accumulation of glutamate [7,25]. In an animal model of capsaicin-induced prostatitis, the injection of BoNT type A was shown to inhibit cyclo-oxygenase-2 expression and suppressed inflammation [26]. In diabetic rats with bilateral allodynia, unilateral subcutaneous injection of BoNT type A in the allodynic region of one affected limb improved allodynia in both limbs, indicating a central analgesic effect of the toxin [27]. BoNT type A has also been shown to inhibit the release of CGRP from trigeminal ganglia and block its excitatory effects in the brain stem sensory neurons [28,29]. Intrathecal administration of BoNT significantly improved neurogenic detrussor overactivity in a chronic spinal cord injury model, as well as pain and bladder inflammation in a cystitis model in rats [30,31].

Several experiments on capsaicin-induced pain in human volunteers have demonstrated the anti-nociceptive properties of BoNTs [28,32]. In humans, prospective RCTs with BoNTs (primarily with onaA) have also demonstrated its efficacy in chronic migraine, post-herpetic, post-traumatic and trigeminal neuralgias, as well as diabetic neuropathy, lateral epicondylitis, plantar fasciitis, and pelvic pain [33].

Recent literature indicates that aboA can also relieve pain in human subjects. In a double-blind study of 24 patients with chronic anterior knee pain due to quadriceps imbalance, injection of 500 units of aboA into the vastus lateralis muscle reduced pain significantly (compared to placebo) at 12 weeks post-injection (*p* < 0.03) [34]. In another study, injection of 40 and 80 units of aboA into the subcutaneous tissue of 50 patients around a painful anal fissure relieved pain at Day 7 post-injection in 82% of the patients [35]. In another study [36], injection of 500 units of aboA into the neck muscles of patients with cervical dystonia resulted in alleviation of neck and shoulder pain in 66% and 76% of the subjects at Weeks 4 and 12. Ranaux et al., in a double-blind study, compared Tsui scores with Toronto Western Spasmodic Torticollis Rating Scale (TWSTRS) pain scores in 54 subjects with cervical dystonia treated with onaA and aboA. Both 3-fold and 4-fold dilutions of aboA alleviated the pain of cervical dystonia better than onaA (*p* = 0.02) [37].

The efficacy of botulinum toxins in low back pain has been suggested previously by two studies. Foster et al. [38], in a double-blind, placebo-controlled study, assessed the effect of onaA on 31 patients with chronic unilateral low back pain. A total 200 units of onaA (40 units per each lumbar level) was injected into each of 5 extensor spinae muscles (regardless of the pain level). At 3 weeks, 11 of 15 patients who received onaA (73.3%) experienced greater than 50% pain relief as compared to 4 of 16 (25%) in the saline group (*p* = 0.012). At 8 weeks, 9 of 15 (60%) in the onaA group and 2 of 16 (12.5%) in the saline group noted relief (*p* = 0.009). Repeat OLBPDQ at 8 weeks showed improvement in 10 of 15 (66.7%) in the onaA group vs. 3 of 16 (18.8%) in the saline group (*p* = 0.011). No side effects were reported. The Therapeutic and Technology Assessment Subcommittee of the American Academy of Neurology defined this study as class II, depicting possible efficacy of onaA in chronic low back pain [39].

In another randomized, placebo-controlled, single-blind study of 50 patients [40], 500 units of aboA were injected into the spine extensors at 5 lumbar levels, similar to the above-mentioned study. After 4 weeks, 76% of patients in the aboA group reported pain relief compared to 20% in the saline group (*p* < 0.005). Additionally, greater pain relief was experienced by patients in the aboA group at 8 weeks (64% vs. 12%; *p* < 0.001). Significant functional improvement (a minimum of two-grade improvement between baseline and post-treatment assessments) was also demonstrated in a higher number of patients receiving aboA than in the saline group (68% vs. 12% respectively; *p* < 0.005) by the 8th week.

In contrast to the two aforementioned studies, there was no statistically significant difference in the outcome between unilateral injection of 50 units each of onaA into the quadratus lumborum and psoas muscles of 29 subjects suffering from bilateral pain compared to saline injection into the same group of muscles on the contralateral side. Both injections reduced the pain by 1.5 grades in the VAS at two weeks on each side [41].

The mechanism(s) by which the botulinum neurotoxins can relieve low back pain is subject to discussion. In our view, it is multifactorial, being similar to the action of the toxin in other pain conditions in some, and different in others. For instance, inhibition of the pain transmitter/modulator release from peripheral nerve endings and DRG as well as reducing local inflammation are believed to be the major mechanisms of botulinum neurotoxins’ analgesic effect in post-herpetic, post-traumatic neuralgias and in painful diabetic neuropathy. These mechanisms are also at work in chronic low back pain which like any chronic pain condition involves peripheral and central sensitization of the sensory system as well as local inflammation which can develop around the nerve roots. Different mechanisms are involved on the motor side. Many patients with low back pain have increased tone in paraspinal muscles as well as painful spasms. Botulinum neurotoxins can relieve painful spasms and reduce muscle tone via blockage of acetylcholine release from neuromuscular junction. Furthermore, muscles injected by botulinum toxins often develop atrophy; and, in low back pain associated with a tight compartment (compartment syndrome), reduction of pressure may relieve pain by easing pressure against pain sensitive structures. Finally, proximity of the injected muscles (erector spinae) to the spinal cord (with L1 level injection included in all positive studies) promotes the central analgesic effect of the toxin, albeit not yet proven. The technical design of our current study with aboA was similar to our previous study with onaA in chronic low back pain, which demonstrated favorable results [38]. In both studies, the target was extensor spinae muscles with injections carried out into all 5 lumbar levels regardless of the level of pain. The less favorable results of the current study can be due to a variety of factors. First, the majority of patients in this report had bilateral low back pain. Bilateral low back pain may not be as responsive to BoNT treatment as unilateral low back pain. Second, the dose of the toxin might have been insufficient. Based on our previous study, we chose 500 units of aboA for each side using a 2.5:1 ratio (2.5 units of AboA for every unit of OnaA). The units of the two toxins are not truly comparable. In addition, in this study, we used the proportion of responders with a VAS of <4 as the primary outcome, whereas, in the previous study, the primary outcome was the number of patients with 2 or more degrees of VAS improvement. As noted above, using the two grade improvement in this study showed a trend for significance (*p* = 0.09).

We acknowledge the limitations of our study. This is a small study and a study with a larger cohort may demonstrate different results. Causal heterogeneity of low back pain in our study cohort may also complicate the results. It is conceivable that the cause of chronic low back pain may have some influence on the magnitude of pain response to botulinum toxin treatment. Better localization of the injected muscles with newer techniques such as ultrasound (as sole technique or in addition to EMG) may help improve the results of botulinum toxin [40,42]. A comparison of studies that use different outcome measures is not possible even when they use identical techniques due to potential problems with toxin dose equivalence and heterogeneity in the patient cohorts. Future investigations into the efficacy of BoNTs in chronic low back pain need to include larger cohorts, and each study should focus on a specific and, as far as possible, a pathologically homogeneous cohort.

## 4. Materials and Methods

### 4.1. Study Design

This was an investigator-initiated, randomized, double-blind, parallel design, placebo-controlled study. The study was funded by Ipsen Pharmaceuticals. A sample size of 80 was selected based on an anticipation of up to 25% placebo effect and a previously reported 52% pain relief after administration of onaA in chronic low back pain [38]. The enrollment plan was set for 90 subjects allowing for a 12% drop out rate. Patients were referred to the study by neurologists and orthopedic surgeons of the hospital. During the baseline visit, the subjects were screened by a neurologist for inclusion and exclusion criteria. The exclusion criteria consisted of age <18, pregnancy, previous history of botulinum toxin treatment, low back pain of less than 3 months’ duration, a pain level <4 on VAS, previous surgery of the lumbo-sacral area, and evidence of acute pathology or infection in magnetic resonance imaging. Moreover, patients with known contraindications (hypersensitivity to BoNTs or disease of the neuromuscular junction) were also excluded. All subjects gave their informed consent for inclusion before they participated in the study. The study (NCT02221648) was conducted in accordance with the Declaration of Helsinki, and the protocol (protocol number 1210011010) was approved by the Human Investigation Committee (HIC).

### 4.2. Methods

After obtaining a thorough clinical history from the eligible subjects, a neurological examination was performed on all. Subjects were on a variety of analgesic medications before entering the study, and these medications were not changed for 30 days prior to or at any point during the study.

They were then randomized into either the aboA group or the placebo group. The level of pain was assessed by the VAS and rated from 0 to 10. In addition, patients filled three questionnaires which described their level of functionality and quality of life: the American Chronic Pain Association’s (ACPA) Quality of Life Scale, the Oswestry Low Back Pain Disability Questionnaire (OLBPDQ), and the Short Form-36 (SF-36) (Table 2 and Table 3). During the baseline visit, subjects were injected with aboA or normal saline into the paraspinal extensor muscles (erector spinae, ES) under electromyographic guidance, using a 1.5 inch, 27.5-gauge needle. The strength of the prepared solution of aboA was 50 units/0.1 cc. When the pain was unilateral with no or subtle pain on the contralateral side, injections were carried out unilaterally. In cases of bilateral pain, the erector spinae muscles were injected on both sides. Similar to the study of Foster et al. [17], in order to influence the entire mass of ES in the lumbar area, regardless of the location of pain, the ES muscles were injected at 5 levels: L1, L2, L3, L4, and L5. The total dose for unilateral injection was 500 units (100 units per level); for bilateral injection, 1000 units. The subjects were observed for 30 min after injection to monitor for any vasovagal or hypersensitivity reactions.

Study subjects revisited the clinic again at Weeks 6, 12, and 16 during which physical examination, the VAS scores, and the three questionnaires were reassessed. In addition, during these visits, the subject’s impression of change was recorded using the Patient’s Global Impression of Change (PGIC) scale (Table 3). The PGIC score varies from “much improved” to “much worse” on a 0 to 7 scale. Subjects were contacted at Weeks 4, 8, and 10 telephonically to assess the presence or absence of any side effects. They mailed the results of the VAS and quality of life questionnaires at Weeks 4, 8, and 10. The primary outcome measure was the proportion of patients with VAS scores of <4 in the aboA and placebo groups at Week 6. The secondary outcomes were changes in the scores of those three questionnaires and PGIC at the same time mark.

## Figures and Tables

**Table 1:** Baseline characteristics of aboA and placebo groups.

Group	N	Mean Age	U/B	Female/Male	VAS	OQ	ACPA
aboA	18	51.3 (18–78)	2/16	14/4	7.7	25.4 (13–38)	5.3 (1–8)
Placebo	19	48.6 (27–69)	2/17	10/9	6.8	19.4 (12–28)	6.1 (1–9)

aboA: abobotulinum toxin A; U/B: unilateral/bilateral Pain; VAS: visual analogue pain scale, 0–10; OQ: Oswestry questionnaire: 50 questions on functionality/quality of life each with 5 subsets. Higher scores represent worse function; ACPA: American Chronic Pain Association’s quality of life scale: 1–10; lower grades represent worse function.

**Table 2:** Number of responders for each scale represented under abobotulinum toxin A and saline columns.

Scale	Abobotulinum Toxin A (*n* = 18)	Saline (*n* = 19)	*p* Value
VAS (4 weeks)	7 (40%)	4 (22%)	*p* = 0.0084
VAS (6 weeks)	5 (28%)	2 (10.5%)	*p* = 0.4470
OWQ	10 (55%)	4 (22%)	*p* = 0.0448
PGIC	8 (44%)	2 (10.5%)	*p* = 0.0293
ACPA	5 (28%)	1 (1%)	*p* = 0.00897

VAS: visual analogue scale; OWQ: Oswestry questionnaire; PGIC: patient global impression of change; ACPA: American Chronic Pain Association’s quality of life scale.

**Table 3:** Study diagram.

	Week 0	Week 4	Week 6	Week 8	Week 10	Week 12	Week 14	Week 16
	Visit 1	Telephone	Visit 2	Telephone	Telephone	Visit 3	Telephone	Visit 4
Eligibility, consent	x							
History */* physical	x		x			x		x
VAS	x	x	x	x	x	x	x	x
ACPA’s QoL scale	x		x			x		x
SF-36	x		x			x		x
PGIC		x	x	x	x	x	x	x
Oswestry Scale	x		x			x		x
Side effectmonitoring		x	x	x	x	x	x	x
Injection	x							

VAS: visual analogue scale; ACPA’s QoL scale: American Chronic Pain Association’s Quality of Life Scale; SF-36: Short Form-36 quality of life questionnaire; Oswestry scale: Oswestry Low Back Pain Disability Questionnaire; PGIC: patient global impression of change. x represents the visit when each of the items in the list was performed.

## References

[1] Murray C.J., Atkinson C., Bhalla K., Birbeck G., Burstein R., Chou D., Dellavalle R., Danaei G., Ezzati M., Fahimi A. (2013). The state of us health, 1990–2010: Burden of diseases, injuries, and risk factors. JAMA.

[2] Hoy D., March L., Brooks P., Blyth F., Woolf A., Bain C., Williams G., Smith E., Vos T., Barendregt J. (2014). The global burden of low back pain: Estimates from the global burden of disease 2010 study. Ann. Rheum. Dis..

[3] Baron R., Binder A., Attal N., Casale R., Dickenson A.H., Treede R.D. (2016). Neuropathic low back pain in clinical practice. Eur. J. Pain.

[4] Balague F., Mannion A.F., Pellise F., Cedraschi C. (2007). Clinical update: Low back pain. Lancet.

[5] Dagenais S., Caro J., Haldeman S. (2008). A systematic review of low back pain cost of illness studies in the united states and internationally. Spine J..

[6] Fiore P., Panza F., Cassatella G., Russo A., Frisardi V., Solfrizzi V., Ranieri M., Di Teo L., Santamato A. (2011). Short-term effects of high-intensity laser therapy versus ultrasound therapy in the treatment of low back pain: A randomized controlled trial. Eur. J. Phys. Rehabil. Med..

[7] Cui M., Khanijou S., Rubino J., Aoki K.R. (2004). Subcutaneous administration of botulinum toxin A reduces formalin-induced pain. Pain.

[8] Aoki K.R., Francis J. (2011). Updates on the antinociceptive mechanism hypothesis of botulinum toxin a. Parkinsonism Relat. Disord..

[9] Rummel A. (2015). The long journey of botulinum neurotoxins into the synapse. Toxicon.

[10] Rossetto O., Pirazzini M., Montecucco C. (2014). Botulinum neurotoxins: Genetic, structural and mechanistic insights. Nat. Rev. Microbiol..

[11] Meng J., Wang J., Lawrence G., Dolly J.O. (2007). Synaptobrevin I mediates exocytosis of cgrp from sensory neurons and inhibition by botulinum toxins reflects their anti-nociceptive potential. J. Cell Sci..

[12] Kitamura Y., Matsuka Y., Spigelman I., Ishihara Y., Yamamoto Y., Sonoyama W., Kamioka H., Yamashiro T., Kuboki T., Oguma K. (2009). Botulinum toxin type A (150 kDa) decreases exaggerated neurotransmitter release from trigeminal ganglion neurons and relieves neuropathy behaviors induced by infraorbital nerve constriction. Neuroscience.

[13] Guo B.L., Zheng C.X., Sui B.D., Li Y.Q., Wang Y.Y., Yang Y.L. (2013). A closer look to botulinum neurotoxin type a-induced analgesia. Toxicon.

[14] Lucioni A., Bales G.T., Lotan T.L., McGehee D.S., Cook S.P., Rapp D.E. (2008). Botulinum toxin type A inhibits sensory neuropeptide release in rat bladder models of acute injury and chronic inflammation. BJU Int..

[15] Wu C., Xie N., Lian Y., Xu H., Chen C., Zheng Y., Chen Y., Zhang H. (2016). Central antinociceptive activity of peripherally applied botulinum toxin type A in lab rat model of trigeminal neuralgia. SpringerPlus.

[16] Matak I., Riederer P., Lackovic Z. (2012). Botulinum toxin’s axonal transport from periphery to the spinal cord. Neurochem. Int..

[17] Gronseth G., French J. (2008). Practice parameters and technology assessments: What they are, what they are not, and why you should care. Neurology.

[18] Xiao L., Mackey S., Hui H., Xong D., Zhang Q., Zhang D. (2010). Subcutaneous injection of botulinum toxin A is beneficial in postherpetic neuralgia. Pain Med..

[19] Wu C.J., Lian Y.J., Zheng Y.K., Zhang H.F., Chen Y., Xie N.C., Wang L.J. (2012). Botulinum toxin type A for the treatment of trigeminal neuralgia: Results from a randomized, double-blind, placebo-controlled trial. Cephalalgia.

[20] Ranoux D., Attal N., Morain F., Bouhassira D. (2008). Botulinum toxin type A induces direct analgesic effects in chronic neuropathic pain. Ann. Neurol..

[21] Konno S., Kikuchi S., Nagaosa Y. (1994). The relationship between intramuscular pressure of the paraspinal muscles and low back pain. Spine.

[22] Peng B., Wu W., Hou S., Li P., Zhang C., Yang Y. (2005). The pathogenesis of discogenic low back pain. J. Bone Joint Surg. Br..

[23] Harrington J.F., Messier A.A., Bereiter D., Barnes B., Epstein M.H. (2000). Herniated lumbar disc material as a source of free glutamate available to affect pain signals through the dorsal root ganglion. Spine.

[24] McLachlan E.M., Janig W., Devor M., Michaelis M. (1993). Peripheral nerve injury triggers noradrenergic sprouting within dorsal root ganglia. Nature.

[25] Luvisetto S., Vacca V., Cianchetti C. (2015). Analgesic effects of botulinum neurotoxin type A in a model of allyl isothiocyanate- and capsaicin-induced pain in mice. Toxicon.

[26] Chuang Y.C., Yoshimura N., Huang C.C., Wu M., Chiang P.H., Chancellor M.B. (2008). Intraprostatic botulinum toxin A injection inhibits cyclooxygenase-2 expression and suppresses prostatic pain on capsaicin induced prostatitis model in rat. J. Urol..

[27] Bach-Rojecky L., Salkovic-Petrisic M., Lackovic Z. (2010). Botulinum toxin type A reduces pain supersensitivity in experimental diabetic neuropathy: Bilateral effect after unilateral injection. Eur. J. Pharmacol..

[28] Gazerani P., Pedersen N.S., Staahl C., Drewes A.M., Arendt-Nielsen L. (2009). Subcutaneous botulinum toxin type A reduces capsaicin-induced trigeminal pain and vasomotor reactions in human skin. Pain.

[29] Durham P.L., Cady R., Cady R. (2004). Regulation of calcitonin gene-related peptide secretion from trigeminal nerve cells by botulinum toxin type A: Implications for migraine therapy. Headache.

[30] Coelho A., Oliveira R., Cruz F., Cruz C.D. (2016). Impairment of sensory afferents by intrathecal administration of botulinum toxin A improves neurogenic detrusor overactivity in chronic spinal cord injured rats. Exp. Neurol..

[31] Coelho A., Oliveira R., Rossetto O., Cruz C.D., Cruz F., Avelino A. (2014). Intrathecal administration of botulinum toxin type A improves urinary bladder function and reduces pain in rats with cystitis. Eur. J. Pain.

[32] Tugnoli V., Capone J.G., Eleopra R., Quatrale R., Sensi M., Gastaldo E., Tola M.R., Geppetti P. (2007). Botulinum toxin type A reduces capsaicin-evoked pain and neurogenic vasodilatation in human skin. Pain.

[33] Jabbari B., Machado D. (2011). Treatment of refractory pain with botulinum toxins—An evidence-based review. Pain Med..

[34] Singer B.J., Silbert P.L., Song S., Dunne J.W., Singer K.P. (2011). Treatment of refractory anterior knee pain using botulinum toxin type A (Dysport) injection to the distal vastus lateralis muscle: A randomised placebo controlled crossover trial. Br. J. Sports Med..

[35] Jost W.H., Schrank B. (1999). Chronic anal fissures treated with botulinum toxin injections: A dose-finding study with Dysport(R). Colorectal Dis..

[36] Hefter H., Benecke R., Erbguth F., Jost W., Reichel G., Wissel J. (2013). An open-label cohort study of the improvement of quality of life and pain in de novo cervical dystonia patients after injections with 500 U botulinum toxin A (Dysport). BMJ Open.

[37] Ranoux D., Gury C., Fondarai J., Mas J.L., Zuber M. (2002). Respective potencies of Botox and Dysport: A double blind, randomised, crossover study in cervical dystonia. J. Neurol. Neurosurg. Psychiatry.

[38] Foster L., Clapp L., Erickson M., Jabbari B. (2001). Botulinum toxin A and chronic low back pain: A randomized, double-blind study. Neurology.

[39] Naumann M., So Y., Argoff C.E., Childers M.K., Dykstra D.D., Gronseth G.S., Jabbari B., Kaufmann H.C., Schurch B., Silberstein S.D. (2008). Assessment: Botulinum neurotoxin in the treatment of autonomic disorders and pain (an evidence-based review): Report of the therapeutics and technology assessment subcommittee of the american academy of neurology. Neurology.

[40] Jazayeri S.M., Ashraf A., Fini H.M., Karimian H., Nasab M.V. (2011). Efficacy of botulinum toxin type A for treating chronic low back pain. Anesthesiol. Pain Med..

[41] De Andres J., Adsuara V.M., Palmisani S., Villanueva V., Lopez-Alarcon M.D. (2010). A double-blind, controlled, randomized trial to evaluate the efficacy of botulinum toxin for the treatment of lumbar myofascial pain in humans. Reg Anesthesia. Pain Med..

[42] Alter K.E., Hallett M., Karp B., Lungu C. (2012). Ultrasound-Guided Chemodenervation Procedures: Text and Atlas.

